# International Experiences in Respite Care and Insights for China: A Scoping Review Based on Health System Framework

**DOI:** 10.1093/geroni/igaf122.853

**Published:** 2025-12-31

**Authors:** Yuxuan Ma, Ye-Fan Glavin, Zhen Wu, Linlin Hu

**Affiliations:** Chinese Academy of Medical Sciences & Peking Union Medical College, Dongcheng District, Beijing, China (People’s Republic); Case Western Reserve University, Cleveland, Ohio, United States; Chinese Academy of Medical Sciences & Peking Union Medical College, Dongcheng District, Beijing, China (People’s Republic); Chinese Academy of Medical Sciences & Peking Union Medical College, Dongcheng District, Beijing, China (People’s Republic)

## Abstract

**Background:**

As China faces the rapid aging challenge, integrating global best practices into respite care systems is of great urgency.

**Methods:**

A systematic review was conducted using “Respite Care” and the four-dimension framework of Health System (service delivery, financing and payment, resource supply and regulation) across PubMed, CNKI, and government portals through February 20, 2025. From 483 records, 23 studies were selected and analyzed.

**Results:**

Analyses revealed diverse respite care models in Europe, North America, Australia. Service delivery: The U.S. NFCSP offers adult day care and emergency respite, Denmark’s “All In One” flexible home visits, and employment support initiatives in the UK and Sweden. Financing and payment: Australia’s RRC caps costs under federal funding, Germany splits insurance costs between employers and employees, while the U.S. relies on federal and state funding.

**Resource supply:**

In terms of workforce, Canada’s PRISMA program integrates community-based respite care via case managers, while the U.S. PACE model deploys multidisciplinary teams.

**Regulation:**

The UK’s Care Act and the U.S. Lifespan Respite Care Act ensure quality and accessibility.

**Implications:**

China’s pilot programs in Beijing and Hangzhou suggest future priorities: scaling community respite centers with diversified services; building professional-informal caregiver networks; expanding long-term care insurance coverage through public-private financing; and establishing quality standards with third-party evaluations.

**Conclusion:**

International experiences emphasize four-dimension system synergy for respite care. China’s path forward lies in adapting these insights to its familial care traditions, fostering a government-led, market-engaged, and socially supported respite care model to cope with the surging wave of population aging.

